# Genome-wide mapping of NBS-LRR genes and their association with disease resistance in soybean

**DOI:** 10.1186/1471-2229-12-139

**Published:** 2012-08-09

**Authors:** Yang Jae Kang, Kil Hyun Kim, Sangrea Shim, Min Young Yoon, Suli Sun, Moon Young Kim, Kyujung Van, Suk-Ha Lee

**Affiliations:** 1 Department of Plant Science and Research Institute for Agriculture and Life Sciences, Seoul National University, Seoul, 151-921, South Korea; 2 Plant Genomics and Breeding Institute, Seoul National University, Seoul, 151-921, South Korea

**Keywords:** Genome duplication, NBS-LRR, Soybean, Transcriptome analysis

## Abstract

**Background:**

R genes are a key component of genetic interactions between plants and biotrophic bacteria and are known to regulate resistance against bacterial invasion. The most common R proteins contain a nucleotide-binding site and a leucine-rich repeat (NBS-LRR) domain. Some NBS-LRR genes in the soybean genome have also been reported to function in disease resistance. In this study, the number of NBS-LRR genes was found to correlate with the number of disease resistance quantitative trait loci (QTL) that flank these genes in each chromosome. NBS-LRR genes co-localized with disease resistance QTL. The study also addressed the functional redundancy of disease resistance on recently duplicated regions that harbor NBS-LRR genes and NBS-LRR gene expression in the bacterial leaf pustule (BLP)-induced soybean transcriptome.

**Results:**

A total of 319 genes were determined to be putative NBS-LRR genes in the soybean genome. The number of NBS-LRR genes on each chromosome was highly correlated with the number of disease resistance QTL in the 2-Mb flanking regions of NBS-LRR genes. In addition, the recently duplicated regions contained duplicated NBS-LRR genes and duplicated disease resistance QTL, and possessed either an uneven or even number of NBS-LRR genes on each side. The significant difference in NBS-LRR gene expression between a resistant near-isogenic line (NIL) and a susceptible NIL after inoculation of *Xanthomonas axonopodis* pv. *glycines* supports the conjecture that NBS-LRR genes have disease resistance functions in the soybean genome.

**Conclusions:**

The number of NBS-LRR genes and disease resistance QTL in the 2-Mb flanking regions of each chromosome was significantly correlated, and several recently duplicated regions that contain NBS-LRR genes harbored disease resistance QTL for both sides. In addition, NBS-LRR gene expression was significantly different between the BLP-resistant NIL and the BLP-susceptible NIL in response to bacterial infection. From these observations, NBS-LRR genes are suggested to contribute to disease resistance in soybean. Moreover, we propose models for how NBS-LRR genes were duplicated, and apply Ks values for each NBS-LRR gene cluster.

## Background

R genes are a key component of gene interactions between plants and biotrophic bacteria and often function to regulate resistance to bacterial invasion [1]. Recent studies have proposed the ‘zigzag model’ to describe the resistance of plants in the context of the co-evolution between invader and host [2]. The first phase of plant defense is pathogen molecular pattern-triggered immunity (PTI) by which the immune system of the plant recognizes a broad range of pathogens with conserved molecular patterns, thereby conferring non-host resistance. In the second phase, effector-triggered immunity (ETI) detects effectors injected into the plant cell with the type III secretion system (TTSS) of bacteria. Usually, ETI results in an amplified PTI response, also known as the hypersensitive reaction (HR).

Among the known types of R proteins, the most common are those that contain a nucleotide-binding site and leucine-rich repeat (NBS-LRR) domain [3]. In the *Arabidopsis* Col-0 genome, 149 proteins were predicted to have NBS-LRR domains and about two-thirds of those were included in a subgroup with at least one known R protein or Col-0 ortholog of an R protein [4]. In soybean, although research on R proteins conferring resistance to diverse diseases is somewhat lacking, NBS-LRR genes with a coiled-coil motif (CC-NBS-LRR) were reported to co-segregate with the *Rpg1-b* locus which confers resistance to strains of *Pseudomonas syringae* pv. *glycinea*[5].

Notably, an NBS-LRR gene with Toll/Interleukin-1 Receptor homology (TIR-NBS-LRR) was reported to restrict nodulation in soybean [6]. Although nodulation is a symbiotic interaction rather than an interaction between pathogen and host, this observation may indicate that R genes control microbe entry into soybean plants. Therefore, NBS-LRR genes, which are located throughout the soybean genome, may be involved in recognizing the presence of pathogens and ultimately conferring resistance.

Plant perception of bacterial invasion is thought to be accomplished by the plant surveillance system, which recognizes specific pathogen-generated effectors and triggers the plant cell to undergo HR [2]. Transcriptome analysis is a powerful tool for studying the role of R genes in disease resistance. Recently, the availability of next-generation sequencers, such as the GS-FLX 454 Titanium and Illumina-GA, has enabled the evaluation of expression levels of all predicted genes in a given plant provided that the sequence of the reference genome is available. After the whole genome draft sequence of soybean was made publicly available at Phytozome (http://www.phytozome.net/soybean.php), soybean transcriptome data could be retrieved for all predicted genes [7]. A previous study presented transcriptome profiling of soybean near-isogenic lines (NILs) between bacterial leaf pustule (BLP, caused by *Xanthomonas axonopodis* pv. *glycines* (*Xag*)) –susceptible and –resistant lines and suggested that differential defense-related gene expression in response to *Xag* may contribute to BLP resistance in soybean [8].

In this study, we examined the correlation and co-localization between the number of NBS-LRR genes and the number of disease resistance QTL on each chromosome and surveyed the functional redundancy of disease resistance genes on the recently duplicated regions known to harbor NBS-LRR genes. Moreover, we investigated the effects of *Xag* inoculation on gene expression in time-course experiments with susceptible and resistant NILs, which revealed a significant increase in the expression of NBS-LRR genes located on QTL previously associated with BLP resistance.

## Results

### Distribution of genome-wide NBS-LRR genes and disease resistance QTL

To define the NBS-LRR genes of the soybean genome, we used whole genome information available at Phytozome (http://www.phytozome.net/soybean.php) [7]. Based on the protein family databases, such as Pfam, Panther, KOG, and KEGG, a total of 46,628 mapped genes have been annotated according to the Gmax_109_annotation_info.txt at Phytozome. To retrieve the putative NBS-LRR genes within the soybean genome, the genes with LRR Pfam IDs PF00560, PF07723 and PF07725 were selected and further refined to include genes containing NBS domains (PF00931). Among 1,286 LRR genes identified in soybean, 319 genes were defined as putative NBS-LRR genes. A total of 314 NBS-LRR genes were located on soybean chromosomes (Table 1), whereas an additional 5 NBS-LRR genes were positioned on scaffolds. NBS-LRR genes were further sorted by their N-terminal domains using homology to already classified Arabidopsis NBS-LRR genes and included 116 TIR-NBS-LRRs, 20 CC-NBS-LRRs, and 183 other NBS-LRRs (see Additional file 1).

**Table 1:** The numbers of NBS-LRR genes, disease resistance QTL, and disease resistance QTL within the 2-Mb flanking region of NBS-LRR genes

**Chromosome**	**Number of NBS-LRR genes**	**Number of QTL**	**Number of QTL within 2-Mb flanking region of NBS-LRR genes**
1	20	8	4
2	10	9	5*
3	36	7	7*
4	1	0	0
5	5	3	3*
6	23	8	7*
7	12	0	0
8	15	10	9*
9	11	9	9*
10	3	16	5
11	5	5	0
12	14	3	2*
13	22	15	12*
14	11	5	3*
15	25	4	4*
16	40	19	19*
17	6	9	4
18	32	34	12
19	10	9	4
20	13	2	1
Total	314	175	110
*R*^2^**			0.5196
*P*-value			0.000336

* More than half of the disease resistance QTL co-localized with NBS-LRR genes within the 2-Mb flanking region.

** Regression analyses were done between the number of NBS-LRR genes and the number of QTL within the 2-Mb flanking region of NBS-LRR genes.

**Figure 1:**
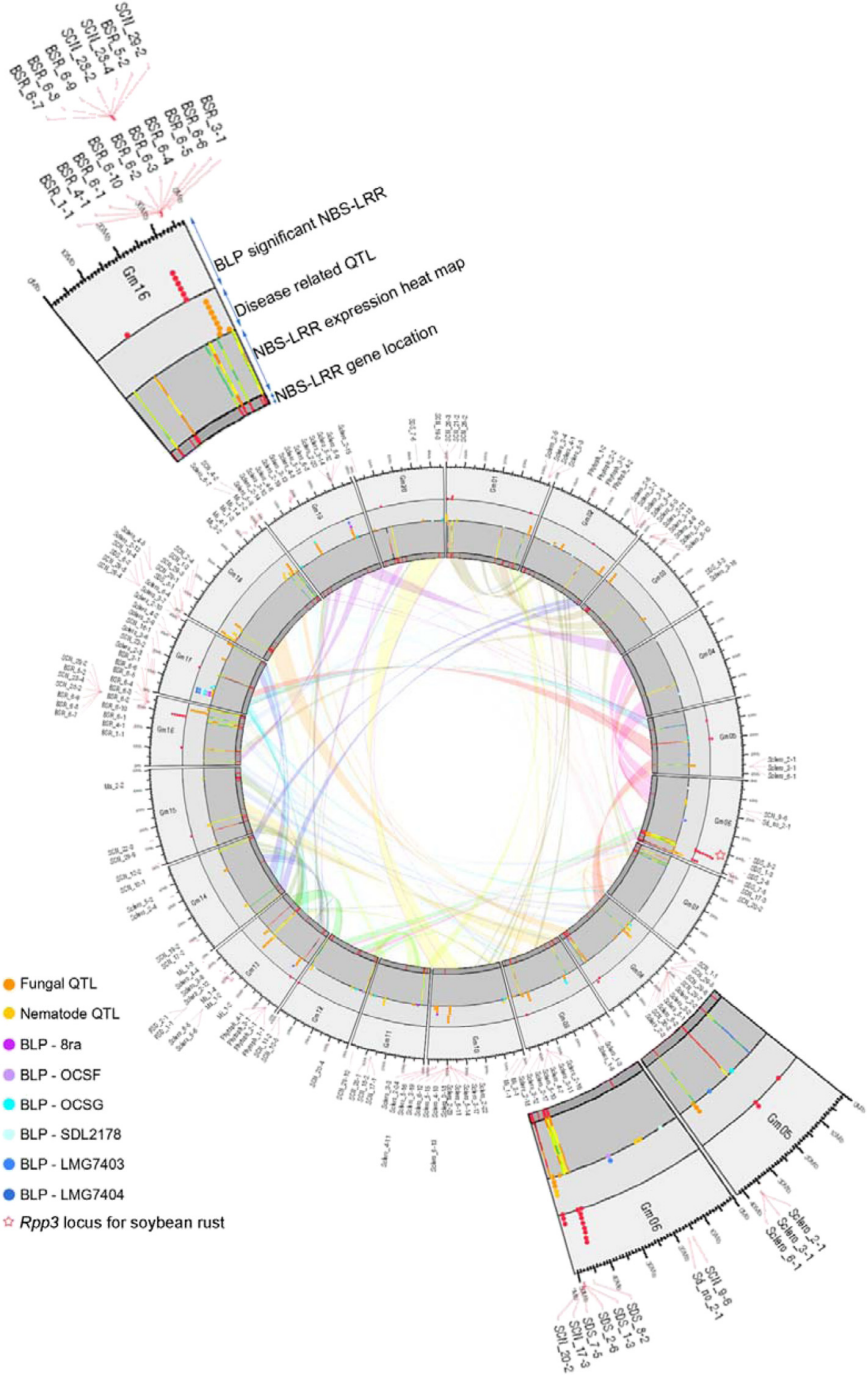
**The circular map showing the locations of recently duplicated regions, locations of NBS-LRR genes, transcription levels of NBS-LRR genes after BLP treatment, locations of disease resistance QTL, and locations of significantly expressed NBS-LRR genes. **For the QTL layer, the dot colors are defined in the legend. On the transcriptome layer, the heatmap shows the expression level of susceptible NIL 0, 6, and 12 hai and resistant NIL 0, 6, and 12 hai from the inner side to the outer side, and the colors were chosen according to the expression level. Minimum values to maximum values of expression are represented with black, grey, red, orange, yellow, lime, green, blue and purple.

To investigate the distribution of NBS-LRR genes in the soybean genome, the chromosomal location of the genes was plotted on a circular genome map at the inner-most layer (Figure 1). The NBS-LRR gene distribution was biased and clustered on chromosomes (Chr) 3, 6, 13, 15, 16, and 18. More than half of the 314 NBS-LRR genes (178) were located on these six chromosomes, suggesting that the NBS-LRR gene clusters are a result of duplication events (Table 1).

Moreover, the disease resistance QTL locations identified using corresponding marker information, were also highly biased and were similar to the locations of the NBS-LRR genes. Among the 296 reported disease resistance QTL on SoyBase (http://soybase.org/), 175 QTL were selected by the corresponding marker information. Figure 1 depicts the location of these disease resistance QTL on the third layer from the innermost layer. Disease resistance QTL tended to be located in the 2-Mb regions flanking the physical locations of the NBS-LRR genes, especially the highly clustered regions (see Additional file 2). For example, the highly clustered region of NBS-LRR genes on Chr 6 harbored 4 QTL conferring fungal resistance and 3 QTL conferring resistance to nematodes. On Chr 16, 40 NBS-LRR genes were clustered, and 14 fungal resistance QTL and 5 nematode resistance QTL were found near the clustered genes. In the case of Chr 18, 8 fungal resistance and 4 nematode resistance QTL were located within the 2-Mb flanking region of a gene cluster containing 32 NBS-LRR genes (Table 1, Figure 1).

Although not all disease resistance QTL were located near NBS-LRR genes, most QTL and NBS-LRR genes tended to co-localize. About 63% of disease related QTL were positioned within the 2-Mb flanking region of an NBS-LRR gene (Table 1). Linear regression analysis revealed that there were significant correlations between the number of NBS-LRR genes and the number of disease resistance QTL within the 2-Mb region flanking an NBS-LRR gene with observed *R*^*2*^ values of 0.520 and a *P*-value lower than 0.001 (Table 1, see Additional file 3). Additionally, QTL for soybean “leaflet shape” traits were selected to understand the correlation between NBS-LRR genes and different types of QTL rather than disease-related (*R*^2^ = 0.073, *P* = 0.251) (see Additional file 4). Moreover, the total of 667 QTL, which were assigned to soybean trait terms, SOY:0000099, (http://soybase.org) was chosen to investigate whether there were other putative effects of NBS-LRR genes (see Additional file 5). From those of selected, a total of 345 QTL were positioned in the 2-Mb flanking regions of NBS-LRR genes and identified that the majority of these traits was soybean morphology and anatomy traits (SOY:0001398) and soybean stress resistance traits (SOY:0001400). The indication showed some NBS-LRR genes could affect many different quantitative traits or in the interaction between disease resistance and other agronomically important traits.

### Recent duplication of NBS-LRR containing genomic regions

To further support the functional correlation between the NBS-LRR genes and disease resistance QTL, we investigated recently duplicated regions in the soybean genome, which could contain genes that perform redundant functions depending on their nature. Figure 1 depicts the recently duplicated region of soybean as a transparent colored ribbon on a circular genome map. Table 2 provides a list of disease resistance QTL within 2 Mb flanking of the recently duplicated regions and NBS-LRR genes within recently duplicated regions were also retrieved. There were a total of 91 NBS-LRR genes within 10 recently duplicated genomic regions and these regions contained the duplicated disease resistance QTL. Among those recently duplicated regions, four regions (IDs: 10176678, 8358327, 11590712, and 10856314) had the same number of NBS-LRR genes. An additional four duplicated regions (IDs: 10176681, 10176680, 18934087 and 18159398) showed a difference of only one gene. In these regions, the copy number of NBS-LRR genes on each side was similar.

**Table 2:** List of NBS-LRR genes on the recently duplicated region and disease resistance QTL in the 2-Mb flanking regions of the recently duplicated regions in soybean

**Recent duplication ID**	**Duplication counterpart**						**Disease resistance QTL**		**NBS-LRR genes**	
	**Chromosome A**			**Chromosome A’**			**Chromosome A**	**Chromosome A’**	**Chromosome A’**	**Chromosome A’**
	**Chr**	**Start**	**End**	**Chr**	**Start**	**End**				
10176681	Gm01	0.97	2.34	Gm09	39.67	40.82	SCN 19-3, SCN 20-3, SCN 21-2	Sclero 2-17, Sclero 3-12	Glyma01g01400, Glyma01g01420	Glyma09g34360, Glyma09g33570, Glyma09g34380
10176680	Gm01	5.04	13.07	Gm02	9.94	12.69	SCN 26-2	SDL2178, Phytoph 1-2	Glyma01g08640, Glyma01g05710	Glyma02g12300
10176678	Gm01	48.26	54.67	Gm11	1.06	6.86	Sclero 2-5, Sclero 3-4, Sclero 4-1, Sclero 5-3	8ra	Glyma01g37620, Glyma01g39000, Glyma01g39010	Glyma11g06260, Glyma11g03780, Glyma11g07680
22835159	Gm03	35.08	47.75	Gm19	37.95	50.53	Sclero 3-16, SDS 8-3	8ra, OCS-F, OCS-G, LMG7403, Sclero 2-20, Sclero 3-14, Sclero 5-12, Sclero 6-9	Glyma03g29270, Glyma03g29370	Glyma19g32150, Glyma19g32090, Glyma19g32110, Glyma19g32180, Glyma19g32080
8358327	Gm08	5.50	11.35	Gm05	30.61	37.28	OCS-G, Sclero 2-2, Sclero 3-2, Sclero 5-1, Sclero 6-2, SCN 29-5, SCN 29-6, SCN 29-7, SCN 30-3, SCN 1-1	LMG7403, Sclero 2-1, Sclero 3-1, Sclero 6-1	Glyma08g12990	Glyma05g29880
18934088	Gm09	32.76	36.86	Gm16	35.20	37.17	Sclero 2-16, Sclero 3-11, Sclero 4-7, Sclero 5-10	BSR 3-1, BSR 4-1, BSR 5-2, BSR 6-1, BSR 6-10, BSR 6-2, BSR 6-3, BSR 6-4, BSR 6-5, BSR 6-6, BSR 6-7, BSR 6-8, BSR 6-9, SCN 28-2, SCN 28-4, SCN 29-2, SCN 1-2, SCN 5-2	Glyma09g29050	Glyma16g33780, Glyma16g33920, Glyma16g34070, Glyma16g33910, Glyma16g34030, Glyma16g33680, Glyma16g32320, Glyma16g33610, Glyma16g34090, Glyma16g33590, Glyma16g33950, Glyma16g34000, Glyma16g33930, Glyma16g34110, Glyma16g33940
18934087	Gm09	42.53	46.38	Gm18	53.56	59.09	Sclero 2-18	Sclero 2-14, Sclero 3-10, Sclero 4-6, Sclero 5-9, Sclero 6-7, Mi 3-2, Mi 4-1, Mi 1-3, Mi 1-4, Mi 2-2, SCN 4-2	Glyma09g39410	Glyma18g46100, Glyma18g46050
18159398	Gm15	39.83	44.16	Gm13	28.75	30.20	Ma 2-2	Sclero 2-12, Sclero 3-9, Sclero 4-4, Sclero 5-6, Sclero 6-5, Ma 1-2, Mj 1-4	Glyma15g37080, Glyma15g36990, Glyma15g35920, Glyma15g37140, Glyma15g37790, Glyma15g35850, Glyma15g37340, Glyma15g37320, Glyma15g37290, Glyma15g36940, Glyma15g36930, Glyma15g37390, Glyma15g37310	Glyma13g25920, Glyma13g26380, Glyma13g26460, Glyma13g25950, Glyma13g25750, Glyma13g26250, Glyma13g26530, Glyma13g25970, Glyma13g26420, Glyma13g25780, Glyma13g26000, Glyma13g26310, Glyma13g26140, Glyma13g26230
11590712	Gm17	39.27	41.04	Gm14	5.85	7.94	Sclero 2-10, Sclero 3-7, Sclero 6-4	SCN 17-2, SCN 19-2	Glyma17g36400, Glyma17g36420	Glyma14g08700, Glyma14g08710
10856314	Gm20	33.04	44.67	Gm10	39.46	50.28	OCS-G, SDL2178, SDS 7-6	8ra, Sclero 2-22, Sclero 2-23, Sclero 2-24, Sclero 3-17, Sclero 3-18, Sclero 3-19, Sclero 4-10, Sclero 4-11, Sclero 5-14, Sclero 5-15, Sclero 5-16, Sclero 6-11, Sclero 6-12, Sclero 6-13	Glyma20g33530, Glyma20g33740, Glyma20g34860	Glyma10g32780, Glyma10g32800, Glyma10g34060

* Chromosome A and A’ represent the recently duplicated region.

** Fungal resistance QTL: *Sclerotinia* stem rot (Sclero), Sudden death syndrome (SDS), Brown stem rot (BSR), *Phytophthora sojae* infection (Phytoph); Nematode resistance QTL: Soybean cyst nematode (SCN), Peanut root-knot nematode (Ma), Southern root-knot nematode (Mi), Javanese root-knot nematode (Mj); Bacterial leaf pustule resistance QTL (BLP): OCS-G, SDL2178, 8ra, OCS-F, LMG7403.

Among these eight duplicated regions, 10176681, 10176680, 10176678 and 11590712 had disease resistance QTL with distinct specificities for divergent diseases (Table 2). For example, the recently duplicated region, 10176681, had duplicated the NBS-LRR genes, Glyma01g01400 and Glyma01g01420 for Chr 1 and Glyma09g33570, Glyma09g34360 and Glyma09g34380 for Chr 9. The QTL for nematode resistance resides on Chr 1, while the QTL for fungal resistance is on Chr 9. These examples indicate that the NBS-LRR genes probably retained their biotic resistance functions after duplication, although they would probably have acquired novel specificities as well. The other duplicated regions (8358327, 10856314, 18934087 and 18159398) had disease resistance QTL that targeted the same disease agents and other diseases as well.

However, two recently duplicated regions (22835159 and 18934088) had a distinct number of NBS-LRR genes, which may have resulted from a tandem duplication occurring independently on one side of the duplicated region. One side of these duplicated regions contained a higher number of NBS-LRR genes and additional or different source of disease resistance QTL compared to the other side. For example, the recently duplicated region, 18934088, has only one duplicated NBS-LRR gene (Glyma09g29050) on Chr 9 and 15 tandemly duplicated genes (Glyma16g32320 to Glyma16g34110) on Chr 16. Several disease resistances QTL also segregate as follows: 4 QTL for resistance to Sclerotinia (Sclero) are located on Chr 9, while 13 QTL for resistance to brown stem rot (BSR) of fungi and 5 QTL for resistance to soybean cyst nematode (SCN) are found on Chr 16.

The BLP resistance QTL that provides bacterial resistance against *Xag* also overlapped with the recently duplicated NBS-LRR regions. A recently duplicated region on Chr 8 (ID 8358327) has two NBS-LRR genes, Glyma08g12990 and Glyma05g29880, on each side (Table 2). The disease resistance QTL that provides resistance against the OCS-G isolate of BLP and against Sclero, was located on one side of the duplicated regions, while the QTL that provides resistance against the LMG7403 isolate of BLP and Sclero was located on the other side of the duplicated region [9]. Another example of a recently duplicated region (ID 10856314), which harbored NBS-LRR genes on both sides, contained a BLP resistance QTL against OCS-G, SDL2178 and SDS 7-6 isolates on one side of the duplicated region and a disease resistance QTL against the 8ra isolate of BLP and Sclero on the other side of the duplicated region.

**Figure 2:**
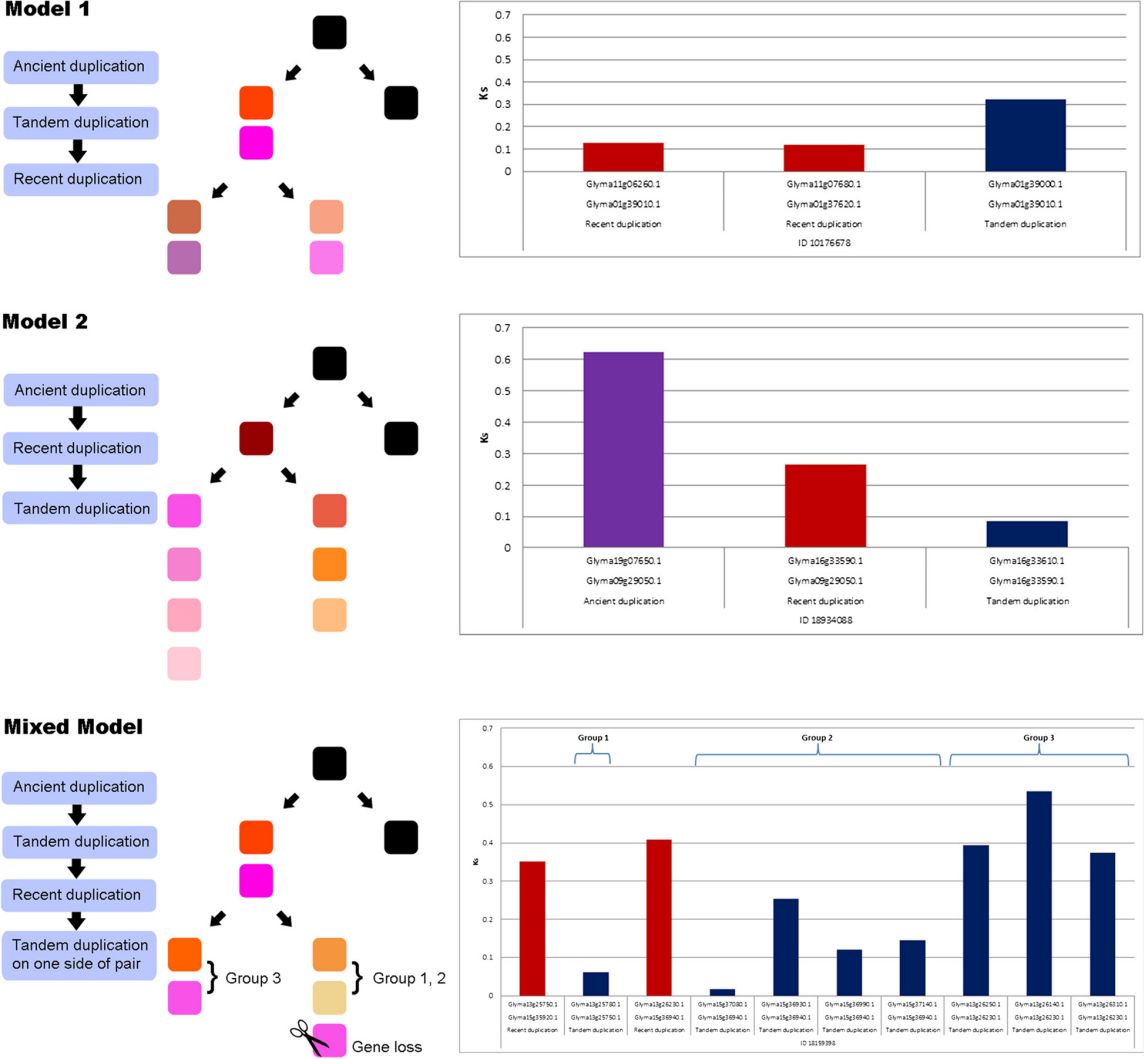
**Models explaining the duplication process of NBS-LRR genes. **In Model 1, the tandem duplication occurred prior to the recent duplication. In Model 2, the ancient duplication event occurred first, the recent duplication event copied a gene or region to another chromosome, and the tandem duplication occurred independently. In the Mixed Model, the tandem duplication occurred prior to the recent duplication and the independent tandem duplication occurred again after the recent duplication. A bar graph of Ks values between recent duplication regions is shown in the right column of the figure. ID 10176678 was matched with Model 1, where tandem duplication occurred prior to the recent duplication. ID 18934088 was matched with Model 2, where an ancient duplication occurred first, followed by a recent duplication and then tandem duplication. ID 18159398 was matched with the Mixed Model; the tandem duplication of Group 3 on chromosome 13 occurred first and the recent duplication followed prior to the independent tandem duplication of Groups 1 and 2, which occurred on chromosomes 13 and 15.

The NBS-LRR genes in recently duplicated regions showed variable synonymous substitution rates (Ks) ranging from 0.016 to 3.722. These values may reflect the order of each duplication event, and herein we propose models to explain the order of duplications. Two models are suggested to explain the duplication orders (Figure 2). In the first model, the NBS-LRR genes were first tandemly duplicated, then subjected to selective pressures over a significant evolutionary time period, and finally were copied to another chromosome. Genes adhering to this model would show high Ks values between tandemly duplicated genes, but low Ks values between paralogs of recently duplicated regions. In the second model, a single NBS-LRR gene was duplicated to another chromosome first and then tandemly duplicated independently. This would yield a pattern that would be the reverse of the first model.

To determine which of these two models is more likely, we estimated the order of the recent duplication and tandem duplication events by estimating the Ks values between paralogs and between the tandemly duplicated regions (see Additional file 6). The paralogs of recently duplicated regions of ID 10176681 and 10176678 showed average Ks values of 0.17 and 0.12, respectively, which are similar to the values reported for recently duplicated paralogs (Ks ≈ 0.13) [7]. Tandemly duplicated regions showed a higher Ks value; hence, we infer that these two regions underwent the duplication according to the first model. Notably, on a recently duplicated region (ID 18934088), we found an anciently duplicated region that showed a Ks value of 0.62, which is consistent with that the value in a previous report (Ks ≈ 0.59) [7]. The Ks values of the paralogous region and tandemly duplicated region were 0.26 and 0.08, respectively. This region might have undergone a duplication process in accordance with the second model after an ancient duplication event. The paralog of ID 22835159 had a Ks value of 0.35 and the tandem duplicated regions had Ks values of 0.50 for one region and 0.16 for the other two regions, suggesting a mixture of the two models. The duplication region ID 18159398 had Ks values of 0.35 and 0.40 for each of the paralogous regions; 0.06 for the tandemly duplicated region on Chr 13 (Group 1); an average of 0.13 on Chr 15 (Group 2); and an average of 0.43 on Chr 13 (Group 3) (Figure 2). The greater Ks value of Group 3 than that of Groups 1 and 2 support a mixed model in which the tandem duplication of Group 3 occurred first, followed by the recent duplication between Chr 13 and Chr 15, and then finally the tandem duplication of Groups 1 and 2.

### Transcriptome analysis of NBS-LRR genes

To explore the disease resistance functions of the NBS-LRRs further, we took advantage of previously generated transcriptome data for BLP-treated NILs [8]. The locations of NBS-LRR genes, which were differentially expressed between the resistant NIL and the susceptible NIL, are shown on the circle map (Figure 1). In total, 35 NBS-LRR genes scattered over 12 chromosomes (Chrs 1, 2, 5, 6, 8, 9, 12, 13, 15, 16, 17 and 20) showed significant expression changes between the resistance and susceptible NILs. Among 35 NBS-LRR genes, five NBS-LRR genes were clustered together at the end of Chr 6 with sudden death syndrome (SDS) and SCN resistance QTL. Additionally, the soybean rust resistance locus, *Rpp3*, were reported to be located around this region (Figure 1) [10] (Table 3). Notably, two NBS-LRRs were clustered on Chr 5, and the BLP resistance QTL was also located in the 2-Mb flanking region.

**Table 3:** **List of thirty-five NBS-LRR motif-containing soybean genes showing a significant expression change between resistant NIL and susceptible NIL in hours after *Xag *inoculation (hai)**

**Genename**	**Description**	**Log**_**2 **_**(fold change)**			**QTL in proximity**	**Recent duplication ID**
		**0 hai**	**6 hai**	**12 hai**		
Glyma01g01420.1	RPM1 (RESISTANCE TO P. SYRINGAE PV MACULICOLA 1); nucleotide binding / protein binding	0.00	0.01	19.28	SCN	10176681
Glyma01g04000.1	disease resistance protein (TIR-NBS-LRR class), putative	−3.11	−2.24	−0.92	No	
Glyma01g04240.1	disease resistance protein (NBS-LRR class), putative	3.10	1.27	0.40	No	
Glyma01g39000.1	disease resistance protein (CC-NBS-LRR class), putative	3.18	−0.81	0.09	No	10176678
Glyma02g32030.1	disease resistance protein (NBS-LRR class), putative	−3.16	1.34	−0.53	No	
Glyma05g09440.1	disease resistance protein (CC-NBS-LRR class), putative	2.62	0.52	0.24	No	
Glyma05g17460.1	disease resistance protein (CC-NBS-LRR class), putative	2.30	0.14	0.17	OCS-G	
Glyma05g17470.1	disease resistance protein (CC-NBS-LRR class), putative	−0.21	0.07	5.31	OCS-G	
Glyma06g39960.1	disease resistance protein (TIR-NBS-LRR class), putative	2.50	0.50	−0.10	No	
Glyma06g40690.1	disease resistance protein (TIR-NBS-LRR class), putative	3.02	0.55	−0.28	No	
Glyma06g40710.1	disease resistance protein (TIR-NBS-LRR class), putative	3.40	0.12	−0.41	No	
Glyma06g40740.1	disease resistance protein (TIR-NBS-LRR class), putative	4.78	0.20	−0.17	No	
Glyma06g40780.1	disease resistance protein (TIR-NBS-LRR class), putative	2.50	0.21	−0.32	No	
Glyma06g40950.1	disease resistance protein (TIR-NBS-LRR class), putative	3.03	−0.40	−0.18	No	
Glyma06g40980.1	disease resistance protein (TIR-NBS-LRR class), putative	2.77	−0.17	−0.14	No	
Glyma06g41330.1	TAO1 (TARGET OF AVRB OPERATION1); ATP binding / protein binding / transmembrane receptor	2.75	0.30	0.15	SDS	
Glyma06g41430.1	disease resistance protein (TIR-NBS-LRR class), putative	2.16	−0.86	0.21	SDS	
Glyma06g46800.1	RPM1 (RESISTANCE TO P. SYRINGAE PV MACULICOLA 1); nucleotide binding / protein binding	18.79	17.33	19.50	SDS, SCN	
Glyma06g46810.1	RPM1 (RESISTANCE TO P. SYRINGAE PV MACULICOLA 1); nucleotide binding / protein binding	21.53	4.02	6.35	SDS, SCN	
Glyma06g46830.1	RPM1 (RESISTANCE TO P. SYRINGAE PV MACULICOLA 1); nucleotide binding / protein binding	3.01	1.16	4.15	SDS, SCN	
Glyma08g41270.1	disease resistance protein (TIR-NBS-LRR class), putative	2.99	0.61	−0.22	No	
Glyma08g42930.1	RPM1 (RESISTANCE TO P. SYRINGAE PV MACULICOLA 1); nucleotide binding / protein binding	2.55	−0.79	−0.19	No	
Glyma09g34360.1	RPM1 (RESISTANCE TO P. SYRINGAE PV MACULICOLA 1); nucleotide binding / protein binding	1.02	0.37	3.85	Sclero	10176681
Glyma13g03770.1	disease resistance protein (TIR-NBS-LRR class), putative	2.44	−0.25	18.78	Phytoph	
Glyma15g13300.1	disease resistance protein (NBS-LRR class), putative	18.70	−0.25	0.39	No	
Glyma16g10340.1	disease resistance protein (TIR-NBS-LRR class), putative	2.39	0.06	−1.29	No	
Glyma16g25020.1	disease resistance protein (TIR-NBS-LRR class), putative	2.83	−0.38	−0.01	No	
Glyma16g25040.1	disease resistance protein (TIR-NBS-LRR class), putative	2.64	−0.20	0.10	No	
Glyma16g25080.1	disease resistance protein (TIR-NBS-LRR class), putative	2.44	−0.20	0.47	No	
Glyma16g25100.1	disease resistance protein (TIR-NBS-LRR class), putative	2.74	−1.06	−0.20	No	
Glyma16g25140.1	disease resistance protein (TIR-NBS-LRR class), putative	2.68	−0.14	−0.20	No	
Glyma16g25170.1	disease resistance protein (TIR-NBS-LRR class), putative	3.15	−0.56	0.07	No	
Glyma17g21130.1	disease resistance protein (CC-NBS-LRR class), putative	0.86	−1.47	3.06	SCN	
Glyma20g06780.1	disease resistance protein (TIR-NBS-LRR class), putative	20.03	−1.36	−17.33	No	

To validate the transcriptome data from Illumina-GA, we performed qRT-PCR with four NBS-LRR genes and one RIN4-like gene (see Additional files 7 and 8). The expression patterns were consistent with the RNA-Seq RPKM values and qRT-PCR values for each gene. The differences in gene expression between NILs were also confirmed to be significant by qRT-PCR (see Additional file 8), which shows that the transcriptome analysis of the NBS-LRRs was reliable.

## Discussion

### NBS-LRR chromosomal distribution

NBS-LRR genes were distributed on several chromosomes on the soybean genome. Especially, Chr 16 had the highest number of NBS-LRR genes as well as the highest number of disease resistance QTL. The other chromosomes containing a biased number of NBS-LRRs also had a tendency to harbor disease resistance QTL. The number of NBS-LRR genes and the number of disease resistance QTL within the 2-Mb flanking regions of the genes were significantly correlated. A similar correlation has been reported in other plants. NBS profiling to identify and map resistance analogues (RGAs) in apple revealed that 25 out of 43 NBS profiling-derived markers mapped close to a major QTL [11]. Barley RGAs co-segregated with known disease resistance loci [12]. In potato, NBS-LRR genes from a BAC library and 38 resistance loci co-localized with TIR-NBS-LRRs, most of which underlie QTL [13]. Therefore, the association between NBS-LRRs and disease resistance loci or QTL might be a common feature of these crop species.

The QTL located near the NBS-LRR cluster were not specific for one particular disease, but rather for several pathogens and pests (e.g., fungi, bacterial, and nematode), and were often closely associated. Interestingly, bacterial genomes have been reported to possess pathogenicity islands on which functionally similar genes are clustered together [14]. These clusters are thought to result from evolution of the mechanisms involved in invasion strategy. The major driving force behind this evolution would be tandem duplication and mobile DNA-like transposases and integrases [15,16]. Notably, *Mutator*-like element (MULE) transposase and the *MuDR* (*Mutator* autonomous element) family transposase domain was located within the NBS-LRR gene, Glyma16g23790, on Chr 16. Moreover, this region of Glyma16g23790 had the highest number of clustered NBS-LRR genes. This finding is consistent with that of a previous study on peanut, which found a high density of transposable elements in BAC clones that contained NBS-LRR genes. These transposable elements might be responsible for the intra-chromosomal duplication and broadening of host specificity [17].

### Recent duplication of the soybean genome and the redundancy of the disease resistance function

Sequencing of the whole soybean genome (var. Williams 82) revealed the actual locations of recently duplicated regions [7]. So far the functional redundancy between duplicated regions has been reported for several traits in soybean including seed protein, oil and BLP resistance [18,19]. According to our analysis, disease resistance was also redundant between recently duplicated regions. Interestingly, some duplicated genome regions contained the same number of NBS-LRR genes, while other duplicated regions had completely different numbers of NBS-LRR genes for both duplicated sides (Table 2).

Sequence divergence of gene families may occur after the duplication process. Specifically, R genes frequently contain numerous copies throughout genome, most likely as a result of unequal crossing over [4,20]. From the proposed models, the possibility of a combination of tandem duplication and inter-chromosomal duplication suggests that the duplication histories of NBS-LRR genes could be one of the factors involved in the diversification of disease-resistant QTL near these genes (Figure 2). However, not all regions with NBS-LRR genes harbored disease-resistant QTL, which might be due to a lack of sufficient disease resistance QTL or to NBS-LRR genes with functions unrelated to disease resistance.

### Transcriptome analysis of the NBS-LRR genes on the BLP-resistant NIL set

The transcriptome analysis of a *Xag*-treated soybean NIL set showed that two NBS-LRR genes with significant fold-changes in expression between the BLP-resistant and the BLP-susceptible NIL were in close proximity to the previously reported BLP-resistant QTL. However, 23 NBS-LRR genes were not near disease resistance QTL. A major gene conferring resistance to BLP on Chr 17 may affect the expression in different types of NBS-LRR genes. It is also possible that RNA-Seq analysis might be sensitive to detect the expression of unrelated genes.

Among several NBS-LRR genes showing significantly different expression levels between resistant and susceptible NILs, six genes (Glyma01g01420, Glyma06g46800, Glyma06g46810, Glyma06g46830, Glyma08g42930 and Glyma09g34360) were annotated as RPM1 (resistance to *Pseudomonas syringae* pv. *maculicola* 1), which was reported to guard the RIN4 protein after its modification by effectors secreted from invading bacteria. Among the one RIN4 gene and four RIN4-like genes identified by the RNA-Seq data, only one (Glyma15g06090.1) showed the log_2_ fold-change of 1.76, between the susceptible and resistant NILs. This gene had a portion of *Arabidopsis* RIN4 and a conserved domain for effector recognition (Table 4). Although the transcriptional profile does not mirror the actual function of the protein, this result suggests that the partial RIN4 gene might be involved in the resistance reaction acting together with RPM1. In tomato, the existence of partial proteins with only an effector-binding domain was proposed as part of a ‘decoy model,’ such as for PTO and RCR3 [21,22].

**Table 4:** List of putative soybean proteins interacting with RPM1 genes containing a cleavage site for pathogenic type III effector (PF05627)

**Gene names**	**Pfam ID**	**Arabidopsis Ortholog**	**Gene descriptions**	**Log**_**2**_**(fold change)**		
				**0 hai**	**6 hai**	**12 hai**
Glyma03g19920.1	PF05627	AT3G25070.1	RIN4 (RPM1 INTERACTING PROTEIN 4);	0.41309	0.090823	0.687695
			protein binding			
Glyma05g31770.1	PF05627	AT5G09960.1	unknown protein	0.695069	−0.09815	−0.01805
Glyma06g15190.1	PF05627	AT5G09960.1	unknown protein	0.322955	0.715957	0.798213
Glyma08g15010.1	PF05627	AT5G09960.1	unknown protein	0.940459	−0.58175	0.072662
Glyma15g06090.1	PF05627	AT5G19473.1	unknown protein	−0.60483	1.116678	1.76209

It is also shown are the fold changes occurring between resistant NIL and susceptible NIL in hourly increments after *Xag* inoculation (hai).

The transcriptomes from several NCBI deposited data were also analyzed to figure out differentially expressed NBS-LRR genes by various disease attacks (see Additional file 9). RNA-Seq data of soybean cultivar Williams 82 inoculated with *Phakopsora pachyrhizi* (MS06-1) (SRP008837, http://www.ncbi.nlm.nih.gov/sra/?term=SRP008837) and cDNA array data of Williams 82 and PI 194639 inoculated with *Sclerotinia sclerotiorum* (GSE15369) were used [23]. Since the soybean symbiotic relationship with *Bradyrhizobium japonicum* is very important for formulating nodules in nitrogen fixation, we also used cDNA array data of SS2-2, supernodulating soybean mutant, response to this bacterium (GSE10340) [24].

Among the differentially expressed NBS-LRR genes in each transcriptome set, Glyma01g04000 was differentially expressed at all four transcriptomes and Glyma05g09440 and Glyma06g40980 were differentially expressed at three transcriptomes, suggesting that those NBS-LRR genes may work against broad range of pathogen attack. Some of NBS-LRR genes had specific responses to different sources of inoculum.

## Conclusions

We analyzed and compared the locations of NBS-LRR genes and disease resistance QTL in the soybean genome to determine the relationship between NBS-LRR genes and resistance functions that have been reported in model plants. The correlation between NBS-LRR genes and disease resistance QTL in the 2-Mb regions flanking NBS-LRR genes was high enough to support the notion that these genes participate in disease resistance. Since the recently duplicated NBS-LRR genes also retained disease resistance QTL, their functional relevance to disease resistance is even more convincing. We also proposed a model for the order of NBS-LRR gene duplications that includes recent duplication and tandem duplication events. Moreover, in the RNA-Seq data of a soybean BLP-resistant and -susceptible NIL set treated with *Xag*, we observed a significant fold-change in NBS-LRR gene expression between the NILs. Two of the differentially expressed NBS-LRR genes were located in the previously reported BLP-resistant QTL region, indicating that NBS-LRR genes might be located downstream of the *Rxp* locus that confers resistance to BLP [25]. By developing molecular markers for NBS-LRR domains, it should be possible to integrate disease resistance against a diverse range of pathogens to generate an elite soybean cultivar.

## Methods

### Data generation

The soybean gene information for the entire genome was retrieved from Phytozome (http://www.phytozome.net/soybean.php as of Dec. 2011). This information contains annotations of domains and a list of *Arabidopsis* homologs and their descriptions [7,26]. To further investigate and define the NBS-LRRs, we used Pfam domain information and the genes with NBS and LRR domains, which were filtered out with the Pfam IDs PF00560, PF07723, and PF07725 for LRR domains and PF00931 for the NBS domain, respectively. Information on recent duplications was also downloaded from Phytozome. The soybean disease resistance QTL were retrieved from SoyBase (http://soybase.org, as of Dec. 2011) for fungal and nematode resistance and from a previous study on BLP-resistance QTL [9]. The physical locations of these QTL were inferred based on the physical location of marker information, which was posted in SoyBase as soybean map version 4.0, and the only QTL with a locatable marker was used for this analysis [27]. The co-localization using linear regression between NBS-LRR genes and disease-related QTL was analyzed in Microsoft Excel.

To define the proximity of NBS-LRR genes, the 2-Mb flanking regions of the genes were considered part of the linkage region, which might affect the QTL statistics. Thus, the 2-Mb flanking region of each of the NBS-LRR genes was retrieved and the QTL included in those regions are depicted in Figure 1. To study the effect of disease resistance redundancy with the recent duplication of the soybean genome, the QTL within the recently duplicated regions and the 2-Mb flanking regions were filtered out. All of this work was implemented with short scripts of Python. The analyzed data were parsed and processed with the Circos software package for visualization [28].

The coding sequences (CDS) for the recently duplicated regions and tandemly duplicated regions were downloaded from Phytozome and the Ks values were calculated with SeqinR, R package software [7,29].

### Transcriptome data analysis

The transcriptome dataset published by Kim et al. regarding BLP-susceptible and BLP-resistant NILs was used [8]. The differentially expressed genes were narrowed down to significantly expressed genes using a cut-off of a four-fold change between the BLP-resistant NIL and the BLP-susceptible NIL. Locations of significantly expressed NBS-LRR genes were placed on the circular map using Circos.

### Validation of RNA-Seq experiments by qRT-PCR

We performed qRT-PCR to validate the RNA-Seq analysis. Four NBS-LRR genes and one RIN4-like gene were tested and primers for these genes were designed with Primer3 software (http://frodo.wi.mit.edu/primer3/) (see Additional files 6 and 7). After cDNA was synthesized using a Bio-Rad iScript™ cDNA Synthesis Kit (Cat. 170-8891, Hercules, CA, USA), synthesized cDNA was subjected to real-time quantification with the LightCycler 480 system (Roche Diagnostics, Laval, QC, Canada) and the Bio-Rad iQ™ SYBR Green Supermix Kit (Cat. 170-8882). PCR mixtures contained 200 ng of cDNA, 500 nM of primer, 18 μl of sterile water, and 25 μl of iQ™ SYBR Green Supermix (Bio-Rad) in total volume of 50 μl. The amplification conditions were 5 min denaturation at 95°C and 40 cycles of 95°C for 10 sec, 60°C for 20 sec and 72°C for 10 sec. After amplification, melting curves were generated in a three-segment cycle of 95°C for 5 sec, 64°C for 1 min, and 97°C for 0 sec in continuous acquisition mode. Quantified expression levels of each target gene were normalized to those of tubulin as a control.

## Authors’ contributions

YJK designed this study and analyzed the sequences using bioinformatics tools. KHK performed RNA-Seq and produced transcriptome data. SRS helped to identify disease resistance QTL from SoyBase. MYY validated the significant gene expressions by qRT-PCR. SS helped with the inoculation of *Xanthomonas axonopodis* pv. *glycines* and confirmed the disease symptoms caused by this bacteria. MYK, KV and SHL helped to design this study as well as draft the manuscript. All authors have read and approved the final manuscript.

## Supplementary Material

Additional file 1 Resistant QTL in the 2-Mb flanking region of CC-NBS-LRRs, TIR-NBS-LRRs and other NBS-LRRs. Click here for file

Additional file 2 List of NBS-LRR regions with QTL in close proximity. Click here for file

Additional file 3 Correlation between the number of NBS-LRR genes and the number of disease resistance QTL within the 2-Mb flanking region of NBS-LRR genes. Click here for file

Additional file 4 The numbers of NBS-LRR genes and Leaflet Shape QTL on each chromosome. Click here for file

Additional file 5 The number of QTL according to trait ontology under 2-Mb flanking regions of NBS-LRR genes. Click here for file

Additional file 6 Ks values of NBS-LRR genes between recently duplicated regions and between tandemly duplicated regions. Click here for file

Additional file 7 Information of qRT-PCR primers used for the confirmation of RNA-Seq analysis. Click here for file

Additional file 8 qRT-PCR validation of RNA-Seq RPKM values with NBS-LRR genes and a RIN4-like gene. Click here for file

Additional file 9 Differentially expressed NBS-LRRs in several experiments. Click here for file
