# Supplementary material for: Genome-wide mapping of NBS-LRR genes and their association with disease resistance in soybean
Source: BMC Plant Biol. 2012 Aug 9;12:139. doi: 10.1186/1471-2229-12-139 (PMC3493331; doi:10.1186/1471-2229-12-139)
Supplement: Additional file 3 — Correlation between the number of NBS-LRR genes and the number of disease resistance QTL within the 2-Mb flanking region of NBS-LRR genes. [file 1471-2229-12-139-S3.docx]

**Additional file 3.** Correlation between the number of NBS-LRR genes and the number of disease resistance QTL within the 2-Mb flanking region of NBS-LRR genes
