# Supplementary material for: Genome-wide mapping of NBS-LRR genes and their association with disease resistance in soybean
Source: BMC Plant Biol. 2012 Aug 9;12:139. doi: 10.1186/1471-2229-12-139 (PMC3493331; doi:10.1186/1471-2229-12-139)
Supplement: Additional file 7 — Information of qRT-PCR primers used for the confirmation of RNA-Seq analysis. [file 1471-2229-12-139-S7.docx]

**Additional file 7.** Information of qRT-PCR primers used for the confirmation of RNA-Seq analysis

| Gene name | Description | Forward primer (5’🡪3’) | Tm (^o^C) | Reverse primer (5’🡪3’) | Tm (^o^C) | Product size (bp) |
| --- | --- | --- | --- | --- | --- | --- |
| Glyma05g09440 | Disease resistance protein  (CC-NBS-LRR class) family | CTCTCTTGGCTTCCGCTCTA | 59.85 | TGGATTCAACCCAACAGTGA | 59.94 | 231 |
| Glyma05g17470 | Disease resistance protein  (CC-NBS-LRR class) family | GCTGGTCCTGGATGATGTCT | 60.08 | ATCTTCGTCGGGAATGTTTG | 59.93 | 217 |
| Glyma06g40740 | Disease resistance protein  (TIR-NBS-LRR class) family | GCCATTGAAGGGTCTCATGT | 59.93 | TCCTTCTCTTGGAACCTGGA | 59.77 | 227 |
| Glyma06g40950 | Disease resistance protein  (TIR-NBS-LRR class), putative | TGAGGCGTTTGGATCTCTCT | 59.95 | ACCGATGGATGGATCAATGT | 60.02 | 269 |
| Glyma06g46810 | RPM1, NB-ARC domain-containing disease resistance protein | GCTAAGAGGCACCCACAAAG | 59.88 | GATGTTGGACCCCATGAATC | 59.99 | 209 |
| Glyma15g06090 | RPM1-interacting protein 4  (RIN4) family protein | GATGGTGTTCACCCAAGCTC | 양식의 맨 위  53.92양식의 맨 아래 | CTTCTTTTTCCCCACGACAA | 양식의 맨 위  53.94양식의 맨 아래 | 187 |
