# Supplementary figures and images for: Genome-wide mapping of NBS-LRR genes and their association with disease resistance in soybean
Source: BMC Plant Biol. 2012 Aug 9;12:139. doi: 10.1186/1471-2229-12-139 (PMC3493331; doi:10.1186/1471-2229-12-139)

| **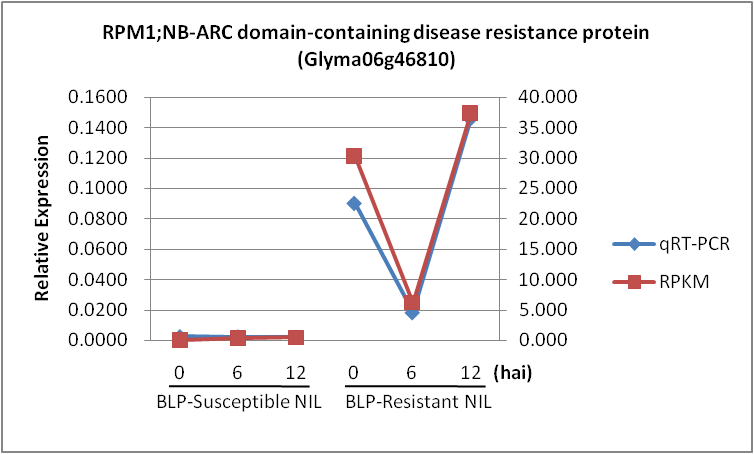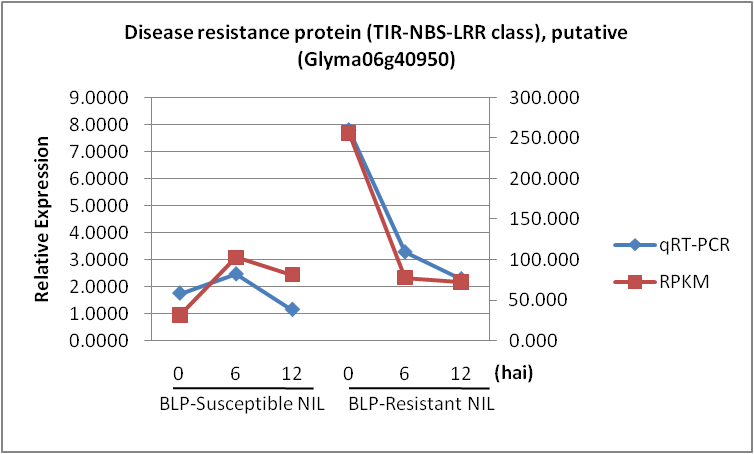** | **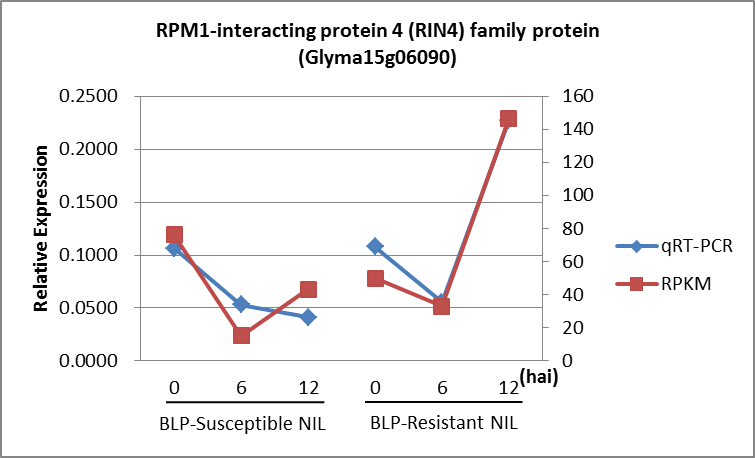**  **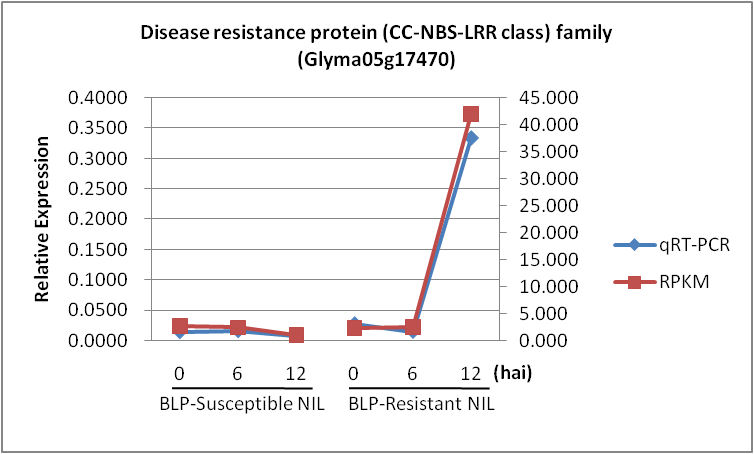** |
| --- | --- |
| **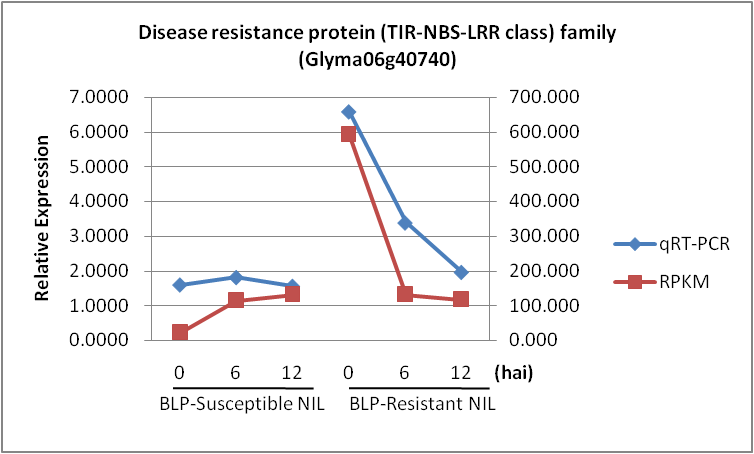** |  |
|  | |

**Additional file 8.** qRT-PCR validation of RNA-Seq RPKM values with NBS-LRR genes and a RIN4-like gene

Supplement: Additional file 8 — qRT-PCR validation of RNA-Seq RPKM values with NBS-LRR genes and a RIN4-like gene. [file 1471-2229-12-139-S8.docx]
